# Isolation of a Novel Reassortant Highly Pathogenic Avian Influenza (H5N2) Virus in Egypt

**DOI:** 10.3390/v11060565

**Published:** 2019-06-18

**Authors:** Naglaa M. Hagag, Ahmed M. Erfan, Mohamed El-Husseiny, Azhar G. Shalaby, Mohamed A. Saif, Maram M. Tawakol, Ahmed A. Nour, Abdullah A. Selim, Abdel-Satar Arafa, Mohamed K. Hassan, Wafaa M. M. Hassan, Hanan A. Fahmy, Essam Ibraheem, Mohamed Attia, Ali M. M. Abdelhakim, Momtaz A. Shahein, Mahmoud M. Naguib

**Affiliations:** 1 National Laboratory for Veterinary Quality Control on Poultry Production, Animal Health Research Institute, 7 Nadi El-Seid Street, 12618 Dokki, Cairo, Dokki-Giza, Egypt; naglaahagagahri@gmail.com (N.M.H.); Ahmed.erfan10000@gmail.com (A.M.E.); olivera_2006@yahoo.com (M.E.-H.); azhargaber0@gmail.com (A.G.S.); mohamed_saif85@hotmail.com (M.A.S.); maram_salah2020@yahoo.com (M.M.T.); mada_boky@yahoo.com (A.A.N.); abdullahselim@yahoo.com (A.A.S.); araby85@hotmail.com (A.-S.A.); mkahassan2020@gmail.com (M.K.H.); fooaaa@live.com (W.M.M.H.); dr.hananfahmy@gmail.com (H.A.F.); essampath61@yahoo.com (E.I.); momtaz.shahein@yahoo.com (M.A.S.); 2 General Organization for Veterinary Services, Nadi El-Said Street P.O. Box, 12619 Dokki, Cairo, Dokki-Giza, Egypt; ashrafatea@yahoo.com (M.A.); Hakam2060@gmail.com (A.M.M.A.); 3 Zoonosis Science Centre, Department of Medical Biochemistry and Microbiology, Uppsala University, Husargatan 3, P.O. Box 582, 75123 Uppsala, Sweden

**Keywords:** Avian influenza, H5N2, H5N8, H9N2, reassortment, poultry, Egypt

## Abstract

Highly pathogenic avian influenza (HPAI) H5N1 and H5N8 have become endemic among domestic poultry in Egypt since 2006 and 2016, respectively. In parallel, the low pathogenic avian influenza H9N2 virus has been endemic since 2010. Despite the continuous circulation of these subtypes for several years, no natural reassortant has been detected so far among the domestic poultry population in Egypt. In this study, the HPAI (H5N2) virus was isolated from a commercial duck farm, giving evidence of the emergence of the first natural reassortment event in domestic poultry in Egypt. The virus was derived as a result of genetic reassortment between avian influenza viruses of H5N8 and H9N2 subtypes circulating in Egypt. The exchange of the neuraminidase segment and high number of acquired mutations might be associated with an alteration in the biological propensities of this virus.

## 1. Introduction

Highly pathogenic avian influenza (HPAI) H5N1 and low pathogenic avian influenza (LPAI) H9N2 viruses have become endemic among domestic poultry in Egypt since 2006 and 2010, respectively [1]. In late 2016, HPAI H5N8 virus of clade 2.3.4.4 (group B) was first reported among wild birds in Egypt [2]. Since 2016, HPAI H5N8 viruses have been reported in different geographical regions across the country in both commercial and backyard bird sectors [3,4], where six genotypes have been detected in both wild and domestic birds [3,4,5]. Moreover, Egypt reported the highest number of human cases with HPAI H5N1 virus worldwide, and one of the only three countries (Egypt, China, and Bangladesh) that reported LPAI H9N2 in humans [1,6]. Recently, simultaneous detection of the three subtypes (H5N1, H5N8, and H9N2) has been described in a poultry farm in Egypt [7]. The co-circulation of those three subtypes increases risks for the generation of reassortants with unpredictable phenotypic properties, including an increased potential threat to human populations. Despite the co-circulation of HPAI H5N1 and LPAI H9N2 viruses for more than eight years, no natural reassortant has been detected so far among domestic poultry population in Egypt between those two subtypes. However, the risk of natural reassortant has been increased after the introduction of the HPAI H5N8 virus in 2016 [1]. This study reports and analyses the genetic and phylogenetic features of the first natural reassortant evidence in domestic poultry in Egypt.

## 2. Materials and Methods

### 2.1. Samples

On 31 December 2018, twenty oropharyngeal and cloacal swabs were collected from a commercial 90-days-old Muscovy duck farm (of 5000 birds) at Mansoura city, Dakahlia governorate, in Egypt. Samples were taken prior to slaughtering as a part of an active surveillance program conducted by the National Laboratory for veterinary quality control on poultry production (NLQP) and General Organization for Veterinary Services. Ducks were vaccinated via an H5N1 vaccine at 7 days old. Ducks were apparently healthy showing no signs of disease. Collected samples were submitted to NLQP for virus identification and isolation. All experiments in this study were conducted in accordance with the ethically approved protocol (AHRI-18032019) of the Animal Health Research Institute, Giza, Egypt.

### 2.2. RNA Extraction and Molecular Diagnosis

Viral RNA was extracted from the pooled samples using the QIAamp Viral RNA Mini Kit (Qiagen, Gmbh, Hilden, Germany) according to the manufacturer’s instructions. Pooled samples were tested using one step Reverse transcription-quantitative polymerase chain reaction (RT-qPCR) (Qiagen, Gmbh, Hilden, Germany) for the M gene of influenza type A viruses [8] using the real-time PCR Mx3005P QPCR System (Agilent, Santa Clara, CA, USA). Positive avian influenza virus (AIV) RNA was subtyped for hemagglutinin (HA) and neuraminidase (NA) using specific subtyping RT-qPCR [9,10].

### 2.3. Virus Isolation

Virus isolation was performed via inoculation into the allantoic cavity of 10-day-old specific pathogen free (SPF) embryonated chicken eggs (ECE) according to the World Organization for Animal Health (OIE) diagnostic manual [11]. Collected allantoic fluid was tested using an hemaglutination assay and specific RT-qPCRs [9,10]. HA-positive allantoic fluids were stored at −80 °C.

### 2.4. Gene Sequencing

The complete genome sequences of the Egyptian virus A/duck/Egypt/VG1099/2018 (EG-VG1099) was amplified using RT-PCR with SuperScript-III One-Step RT-PCR System with Platinum^®^ Taq DNA Polymerase (Invitrogen, Waltham, MA, USA) with primers described previously [12,13]. The gene-specific RT-PCR amplicons were size-separated using agarose gel electrophoresis, excised, and purified from gels using the QIAquick Gel Extraction Kit (Qiagen, Gmbh, Hilden, Germany). Further, purified PCR products were used directly for cycle sequencing reactions using the BigDye Terminator v3.1 Cycle Sequencing Kit (Applied Biosystems, Foster City, CA, USA). Reaction products were purified using Centrisep spin columns (ThermoFisher, Waltham, MA, USA) and sequenced on an ABI 3500XL Genetic Analyzer (Life Technologies, Carlsbad, CA, USA). Sequences generated in this study were submitted to the Global Initiative on Sharing All Influenza Data (GISAID) platform under accession number: EPI1387245-52.

### 2.5. Genetic and Phylogenetic Characterization

The obtained sequences were assembled and edited using the Geneious^®^ software, version 11.0.5 [14]. A Basic Local Alignment Search Tool (BLAST) search was performed using Global Initiative on Sharing All Influenza Data (GISAID) platform, and sequences used in this study have been retrieved from the GISAID database for representative H5N8, H9N2, and other similar viruses. Alignment and identity matrix analyses were done using Multiple Alignment using Fast Fourier Transform (MAFFT) [15]. Phylogenetic analyses were based on a maximum likelihood methodology based on Bayesian (BIC) criterion after selection of the best fit models using IQ-tree software version 1.1.3 [16]. Trees were finally viewed and edited using FigTree v1.4.2 software (http://tree.bio.ed.ac.uk/software/figtree/).

## 3. Results

### 3.1. Virus Isolation

Pooled samples were found positive for the influenza A H5N2 subtype and negative for H7 and H9, as well as N1 and N8. Ducks were apparently healthy with neither disease symptoms nor abnormal mortalities. The virus was isolated, confirmed, and named A/duck/Egypt/VG1099/2018 (EG-VG1099).

### 3.2. Genetic and Phylogenetic Characterization

**Figure 1:**
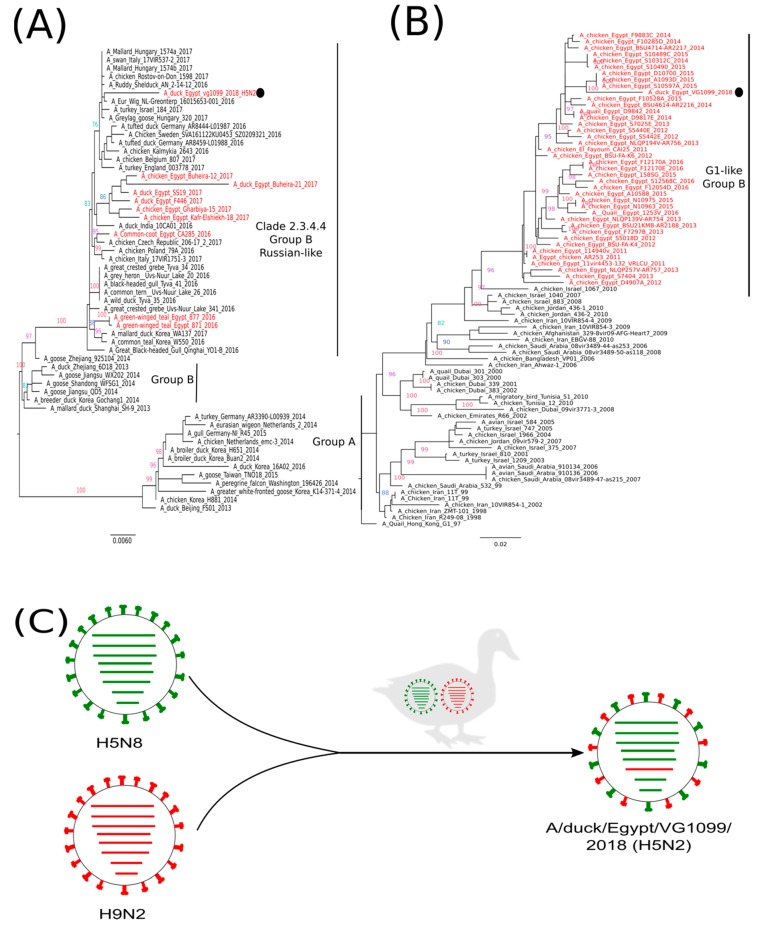
Phylogenetic trees of the nucleotide sequences of the HA (**A**) and NA (**B**) gene segments. Maximum likelihood calculations were done using the IQTree software under the best fit model according to the Bayesian criterion. Egyptian H5N2 virus is indicated with a black dot. (**C**) Emergence and possible reassortment option of the novel HPAI H5N2 virus found in Egypt in 2018.

The complete genome sequences of the Egyptian virus EG-VG1099 were obtained and sequences generated in this study were submitted to the Global Initiative on Sharing All Influenza Data (GISAID) platform under the accession number: EPI1387245-52. Nucleotide identity analysis showed that the EG-VG1099 virus was closely related, according to its hemagglutinin (HA) gene segment, to HPAI H5N8 viruses isolated in Europe 2016–2017, with a nucleotide sequence identity of 98% to the A/Eur_Wig/NL-Greonterp/16015653-001/2016; the same identity of 98% with A/duck/Egypt/F446/2017(H5N8) virus was also recorded. The neuraminidase (NA) gene of the EG-VG1099 virus shared 97% nucleotide identity with the Egyptian LPAI H9N2 virus A/chicken/Egypt/D10700/2015(H9N2) (Table 1), and no identity with any virus outside Egypt in the first 200 hits was observed. Six segments—polymerase basic (PB2), PB1, polymerase acidic (PA), nucleoprotein (NP), matrix protein (M), nonstructural protein (NS)—revealed a nucleotide identity of 98%–99% with the Dutch (A/Eur_Wig/NL-Greonterp/16015653-001/2016) and Italian (A/swan/Italy/17VIR537-2/2017) HPAI H5N8 viruses (Table 1). Possible reassortment option of the novel Egyptian HPAI H5N2 virus is illustrated in Figure 1C.

**Figure 2:**
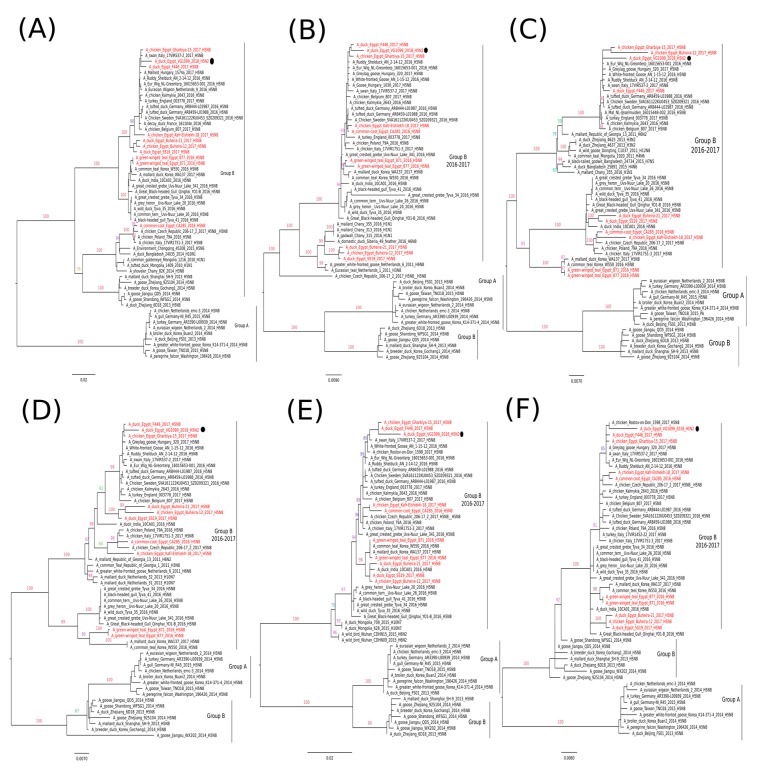
Phylogenetic trees of the nucleotide sequences of the gene segments PB2, PB1, PA, NP, M, and NS (**A**–**F**). Maximum likelihood calculations were done with the IQTree software under the best fit model according to the Bayesian criterion. Egyptian HPAI H5N8 viruses are colored in red and the H5N2 virus is indicated with a black dot.

Phylogenetic analysis of the HA gene segment showed that the EG-VG1099 virus was closely related to HPAI H5N8 viruses reported in Europe during 2016–2017 (2.3.4.4 clade, group B), and phylogenetically distinguished from the previously reported HPAI H5N8 viruses in Egypt (Figure 1A). The NA gene segment of EG-VG1099 was phylogenetically grouped with the LPAI H9N2 viruses isolated from Egypt (Figure 1B). The remaining six genes (PB2, PB1, PA, NP, M, and NS) of the EG-VG1099 virus were clustered with Egyptian HPAI H5N8 viruses: A/chicken/Egypt/Gharbiya-15/2017 and A/duck/Egypt/F446/2017, and revealed a close phylogenetic relatedness with H5N8 viruses frequently found in Europe in 2016–2017 (Figure 2).

The HA gene of the EG-VG1099 virus possessed multiple basic amino acids, “PLREKRRKR/GLF”, in the cleavage site of the HA indicating high pathogenicity of this virus. The EG-VG1099 virus exhibited R76S, S98R, A138S, A160V, and R173Q amino acid substitutions (H3 numbering) at its HA protein, distinguishing it from previously reported HPAI H5N8 viruses of clade 2.3.4.4 (group B). Amino acid substitutions at positions S98R, A138S, and A160V have been reported previously to be related to virulence and host specificity [17,18]. The NA coded protein of the emerged HPAI H5N2 virus was distinguished by 43 nucleotide mutations from the Egyptian LPAI H9N2 viruses (during 2010–2016, no available published sequence hereafter). Twelve out of the 43 were non-synonymous and encoded amino acid substitutions: F37L, T43A, V50E, K143E, R199K, M210I, L211I, R283Q, R288I, R344V, N384K, and K415R. Positions 143 and 344 were located in the antibodies recognition sites of the NA coded protein [19,20]. However, none of the remaining amino acid substitutions have been described in association with resistance to neuraminidase inhibitors or located in the hemadsorption site. Further, the Egyptian EG-VG1099 possessed no mutations at known molecular features associated with virulence or host adaptation, like E627K and D701N in PB2, N375S and L598P in PB1, or V100A in PA [17]. However, N30D and T215A amino acid substitutions in the M1 protein and P42S in the NS1 protein (previously reported in the Egyptian H5N8 viruses) were observed, suggesting that the virus could exhibit increased virulence in mammals [17].

## 4. Discussion

Currently, Egypt faces endemic co-circulation of HPAI (H5N1, H5N8) and LPAI (H9N2) viruses, where a simultaneous detection of the three subtypes has been detected [7]. The co-circulation of those three subtypes raises fears for the generation of a new subtype/genotype with unpredictable properties, including an increased potential threat to human [1]. However, the nucleotide identity and phylogenetic distance of the Egyptian HPAI H5N2 virus (EG-VG1099) virus with other Egyptian HA genes seems low, and it is reasonable to suggest that the new reassortant EG-VG1099 resulted from a reassortant between HPAI H5N8 virus of clade 2.3.4.4 (group B) and the Egyptian LPAI (H9N2) virus of the G1-like lineage (Figure 1C). This was supported by the phylogenetic relatedness of the NA gene segment with only viruses isolated from Egypt; however, the circulation of an additional variant of H5N8 in Egypt cannot be excluded. So far, no natural reassortant has been detected between the Egyptian H5N1/H9N2 subtypes [21], and the emerged H5N2 virus in this study indicated a higher reassortment compatibility between the Egyptian H5N8 and H9N2 viruses compared to the Egyptian H5N1/H9N2. However, we cannot exclude the detection of new genotypes with different gene constellations in the upcoming period. This highlights the significance of obtaining the whole genome sequence of the circulating HPAI H5N1/H5N2/H5N8 viruses in Egypt.

Further, different amino acid substitutions associated with either virulence or host adaptation have been observed in the newly detected Egyptian HPAI H5N2 virus (EG-VG1099). Amino acid substitutions (S98R, A138S, and A160V) in the antibody recognition sites of the HA have been observed. These mutations were described to enhance the binding of AIV H5 with the α2.6 sialic acid receptor and increase virulence in the mammalian animal model [17,18]. Further, N30D and T215A amino acid substitutions in the M1 protein of the EG-VG1099 virus were reported to enhance the virulence of the HPAI H5N1 virus in mice [17]. In addition, P42S in the NS1 protein also has been found to increase the virulence of the HPAI H5N1 virus in mice [17]. Interestingly, a high number of non-synonymous nucleotide mutations (*n* = 12) in the NA have been noticed. Only positions 143 and 344 are located in the antibody recognition sites of the NA coded protein and have been reported in the literature to be related to the antigenic drift/escape mutant [19,20]. However, none of the rest have been described in association with resistance to neuraminidase inhibitors or located in the hemadsorption site. At this point, it cannot be excluded that the new HPAI H5N2 subtype, reported in this study, may be associated with an alternation in the biological propensities. Hence, future studies in birds (e.g., chickens, ducks) and mammalian models (e.g., ferrets, mice) is recommended to provide experimental data on the fitness and virulence of this virus subtype. In addition, in vivo and in vitro studies comparing the newly detected H5N2 virus to a closely related H5N8 virus are required to determine any alteration in the phenotypic properties between the two viruses.

Moreover, our study underlines the importance of active surveillance in the timely detection of new AIV subtypes. It can be expected that continuing surveillance activities might lead to the detection of additional new cases; hence, surveillance should be strengthened with special emphasis in the surrounding regions of the outbreak. Interventions, like improved biosecurity practices in poultry production enterprises, and live bird trading and marketing practices in Egypt, should be considered to reduce the dissemination of AIV and reduce the chance of reassortment raised through co-infection.

## Figures and Tables

**Table 1:** Nucleotide sequence identities between the EG-VG1099 (H5N2) virus and nearest homologues in the GenBank and GISAID database.

Gene	Virus	Subtype	Accession Number	Collection Date	Identity
PB2	A/Ruddy Shelduck/AN/2-14-12/2016	H5N8	EPI1184014	2016-12-14	98%
	A/swan/Italy/17VIR537-2/2017	H5N8	EPI954556	2017-01-19	98%
	A/chicken/Egypt/Gharbiya-15/2017	H5N8	EPI1104288	2017	98%
	A/Eur_Wig/NL-Greonterp/16015653-001/2016	H5N8	EPI1019555	2016-12-08	98%
Pb1	A/White-fronted Goose/AN/1-15-12/2016	H5N8	EPI1184005	2016-12-15	99%
	A/Eur_Wig/NL-Greonterp/16015653-001/2016	H5N8	EPI1019556	2016-12-08	99%
	A/duck/Egypt/F446/2017	H5N8	EPI1018205	2017-04-06	99%
	A/swan/Italy/17VIR537-2/2017	H5N8	EPI954557	2017-01-19	99%
PA	A/Eur_Wig/NL-Greonterp/16015653-001/2016	H5N8	EPI1019554	2016-12-08	98%
	A/White-fronted Goose/AN/1-15-12/2016	H5N8	EPI1184004	2016-12-15	98%
	A/duck/Egypt/F446/2017	H5N8	EPI1018206	2017-04-06	98%
	A/swan/Italy/17VIR537-2/2017	H5N8	EPI954555	2017-01-19	98%
HA	A/Eur_Wig/NL-Greonterp/16015653-001/2016	H5N8	EPI1019558	2016-12-08	98%
	A/swan/Italy/17VIR537-2/2017	H5N8	EPI954559	2017-01-19	98%
	A/White-fronted Goose/AN/1-15-12/2016	H5N8	EPI1183999	2016-12-15	98%
	A/duck/Egypt/F446/2017	H5N8	EPI1018207	2017-04-06	98%
NP	A/chicken/Egypt/Gharbiya-15/2017	H5N8	EPI1104284	2017	99%
	A/duck/Egypt/F446/2017	H5N8	EPI1018208	2017-04-07	99%
	A/Eur_Wig/NL-Greonterp/16015653-001/2016	H5N8	EPI1019551	2016-12-08	99%
	A/swan/Italy/17VIR537-2/2017	H5N8	EPI954552	2017-01-19	99%
NA	A/chicken/Egypt/D10700/2015	H9N2	KX000734*	2015-02-26	97%
	A/chicken/Egypt/S107569A/2015	H9N2	KX000727*	2015-02-15	97%
	A/chicken/Egypt/A1093D/2015	H9N2	KX000717*	2015-02-09	97%
M	A/chicken/Egypt/Gharbiya-15/2017	H5N8	EPI1104286	2017	98%
	A/duck/Egypt/F446/2017	H5N8	EPI1018210	2017-04-06	98%
	A/swan/Italy/17VIR537-2/2017	H5N8	EPI954554	2017-01-19	98%
	A/Eur_Wig/NL-Greonterp/16015653-001/2016	H5N8	EPI1019553	2016-12-08	98%
NS	A/Eur_Wig/NL-Greonterp/16015653-001/2016	H5N8	EPI1019552	2016-12-08	98%
	A/chicken/Egypt/Gharbiya-15/2017	H5N8	EPI1104285	2017	98%
	A/swan/Italy/17VIR537-2/2017	H5N8	EPI954553	2017-01-19	98%
	A/duck/Egypt/F446/2017	H5N8	EPI1018211	2017-04-06	98%

* GenBank accession number.
